# Time-Course of Changes in Photosynthesis and Secondary Metabolites in Canola (*Brassica napus*) Under Different UV-B Irradiation Levels in a Plant Factory With Artificial Light

**DOI:** 10.3389/fpls.2021.786555

**Published:** 2021-12-22

**Authors:** Jin-Hui Lee, Seina Shibata, Eiji Goto

**Affiliations:** ^1^Graduate School of Horticulture, Chiba University, Matsudo, Japan; ^2^Plant Molecular Research Center, Chiba University, Chiba, Japan

**Keywords:** antioxidant capacity, bioactive compounds, environmental stress, phytochemical, microarray

## Abstract

This study aimed to evaluate short-duration (24 h) UV-B irradiation as a preharvest abiotic stressor in canola plants. Moreover, we quantified the expression levels of genes related to bioactive compounds synthesis in response to UV-B radiation. Canola seedlings were cultivated in a plant factory under artificial light (200 μmol m^–2^ s^–1^ photosynthetic photon flux density; white LED lamps; 16 h on/8 h off), 25°C/20°C daytime/nighttime air temperature, and 70% relative humidity. Eighteen days after sowing, the seedlings were subjected to supplemental UV-B treatment. The control plants received no UV-B irradiation. The plants were exposed to 3, 5, or 7 W m^–2^ UV-B irradiation. There were no significant differences in shoot fresh weight between the UV-B-irradiated and control plants. With increasing UV-B irradiation intensity and exposure time, the H_2_O_2_ content gradually increased, the expression levels of genes related to photosynthesis downregulated, and phenylpropanoid and flavonoid production, and also total phenolic, flavonoid, antioxidant, and anthocyanin concentrations were significantly enhanced. The genes related to secondary metabolite biosynthesis were immediately upregulated after UV-B irradiation. The relative gene expression patterns identified using qRT-PCR corroborated the variations in gene expression that were revealed using microarray analysis. The time point at which the genes were induced varied with the gene location along the biosynthetic pathway. To the best of our knowledge, this is the first study to demonstrate a temporal difference between the accumulation of antioxidants and the induction of genes related to the synthesis of this compound in UV-B-treated canola plants. Our results demonstrated that short-term UV-B irradiation could augment antioxidant biosynthesis in canola without sacrificing crop yield or quality.

## Introduction

Phytochemicals are naturally occurring, bioactive, and non-nutrient compounds in plants (Visioli et al., 2011). They include polyphenols, terpenoids, alkaloids, carotenoids, aromatic glucosinolates, among others. Some of these promote human health, as they are anti-inflammatory, anticancer, antioxidant, and so on (Reddy et al., 2003; Scalbert et al., 2011). Research interest in foods containing functional antioxidant phytochemicals has recently grown. The quantity and quality of phytochemicals may be improved through various environmental controls. Plant factories and vertical farms can precisely regulate the ambient environment and are suitable as production systems for crops rich in phytochemicals (Goto, 2011).

Plants control a wide range of physiological processes and use UV radiation as an environmental signal. The three types of UV radiation are UV-C (100–280 nm), UV-B (280–315 nm), and UV-A (315–400 nm) (Madronich et al., 1998). However, only UV-A and UV-B radiations reach the surface of the Earth, as UV-C is absorbed mainly by the tropospheric ozone layer. As UV-B irradiation range is highly energetic and has a short wavelength, it can generate excessive reactive oxygen species (ROS) in plants exposed to high levels of it. Excessive ROS can damage DNA, proteins, membranes, and the photosynthetic apparatus. Hence, they can adversely affect plant growth and development (Tevini et al., 1981; Flint and Caldwell, 1984; Tevini and Steinmüller, 1987; Jenkins, 2009). In contrast, low-level UV-B irradiation promotes morphological responses in plants, such as leaf growth, stomatal differentiation, and the inhibition of hypocotyl elongation. Therefore, the morphophysiological responses of plants to UV irradiation vary with intensity and exposure duration. However, even at the same UV irradiation intensity and exposure time plant responses vary with species, genotype, resistance, sensitivity, leaf thickness, and other factors (Rozema et al., 1997; Kataria et al., 2014; Lee and Oh, 2015). According to a previous report (Sytar et al., 2018), the sensitivity of lettuce to UV was different depending on the species, and the content of secondary metabolites (total phenol, flavonoid, anthocyanin, and phenolic acids) also varied when different lettuce species were exposed to the same UV conditions.

Plants may adapt to augmented UV-B irradiation by increasing secondary metabolite production. UV-B-responsive genes are induced *via* UV resistance locus 8 (UVR8)-dependent UV-B signaling pathway, and promote the accumulation of phenolic compounds, such as hydroxycinnamic, ferulic, caffeic, and sinapic acids and flavonoid compounds, such as luteolin, kaempferol, and quercetin (Jenkins, 2009; Wargent et al., 2009; Neugart et al., 2014; Escobar et al., 2017). The overall responses of plants to UV-B irradiation are governed by acclimatization mechanisms, such as the accumulation of compounds that absorb UV-B radiation, and they protect the photosynthetic apparatus from injury (Allen et al., 1998; Chang et al., 2009). The long-term effects of UV-B irradiation on plants have been extensively investigated, and the findings of these studies helped predict the consequences of increasing UV exposure. Nevertheless, few studies have focused on the impact of short-term UV-B exposure on plants. As the photosynthesis process is sensitive to UV-B irradiation, most plant species are affected by it and their growth may be impaired in response to prolonged UV exposure. Therefore, research is being conducted on increasing the phytochemical content of plants without inhibiting their growth. It was recently discovered that short-term (several days) UV-B irradiation might serve as a preharvest treatment to obtain plant products rich in antioxidants (Pandey and Pandey-Rai, 2014; Inostroza-Blancheteau et al., 2016). Studies on the effects of short-term UV-B irradiation may help elucidate UV-induced signaling pathways and trends in genes and/or parameters that immediately respond to UV irradiation.

Previous research showed that upregulation of the expression of genes related to bioactive secondary metabolites synthesis triggered by UV-B exposure may vary with duration. Inostroza-Blancheteau et al. (2016) found that *PAL*, *CHS*, and *F3’H* expression levels were upregulated within 6 h of UV-B exposure. Elevated total phenolic content was observed in highbush blueberry leaves (*Vaccinium corymbosum* L. cv. Bluegold) exposed to UV-B for 24 h. In addition, Neugart and Bumke-Vogt (2021) investigated the effects on major genes expression of the phenylpropanoid pathway, contents of the flavonoid groups, and hydroxycinnamic acid derivatives after short-term UV-B irradiation and before the harvest of various *Brassica* species (*B. rapa, B. nigra, B. oleracea, B. juncea, B. napus*, and *B. carinata*). The response during the acclimation period after the UV-B irradiation (2 and 24 h) was investigated, but the response immediately after the UV-B irradiation was not confirmed. If we conduct a study to check the changes in gene expression and the content of bioactive compounds immediately after UV exposure, it is expected that the temporal differences between gene expression and the synthesis of bioactive compounds in response to UV irradiation are elucidated.

Hence, this study aimed to identify UV-B treatment conditions conducive to the accumulation of bioactive compounds without inhibiting plant growth. Furthermore, this study was performed to confirm the expression patterns of photosynthesis and secondary metabolites biosynthesis related genes and the increasing patterns of bioactive compounds according to UV-B exposure time in canola plants.

## Materials and Methods

### Plant Materials and Cultivation Conditions

The experiments were conducted at Chiba University, Japan, in a closed plant production system with multilayer cultivation shelves. Canola (*Brassica napus* L. cv. Kizakino-natane) was the plant material. It was used as a model for research on the growth, gene expression, and accumulation of bioactive compounds in a *Brassica* leaf vegetable cultivated under various environmental conditions (Goto et al., 2020; Son et al., 2020). The seeds were germinated on Kimtowels (NIPPONPAPER CRECIA Co. Ltd., Tokyo, Japan). One day after sowing (DAS), the germinated seeds were transplanted to M-size polyurethane sponges (M Hydroponics Laboratory Co. Inc., Aichi, Japan). The seedlings were then transplanted to 18.6-L hydroponic containers (San Box No. 26B; SANKO Co. Ltd., Tokyo, Japan) under white LED lamps (LDL40S-N/19/21; Panasonic Corp., Osaka, Japan) and cultivated until 18 DAS. A quarter-strength Otsuka A formulation (OAT house A treatment; OAT Agrio Co. Ltd., Tokyo, Japan) was the nutrient solution used in all the experiments. The pH and electrical conductivity of the nutrient solution were ∼6.4-6.5 and ∼1.0-1.1 dS m^–1^, respectively. The environmental conditions were 200 μmol m^–2^ s^–1^ photosynthetic photon flux density, 16-h light/8-h dark, 25°C/20°C daytime/nighttime air temperature, 70% RH, and 1,000 μmol mol^–1^ CO_2_.

### UV-B Treatment

**FIGURE 1:**
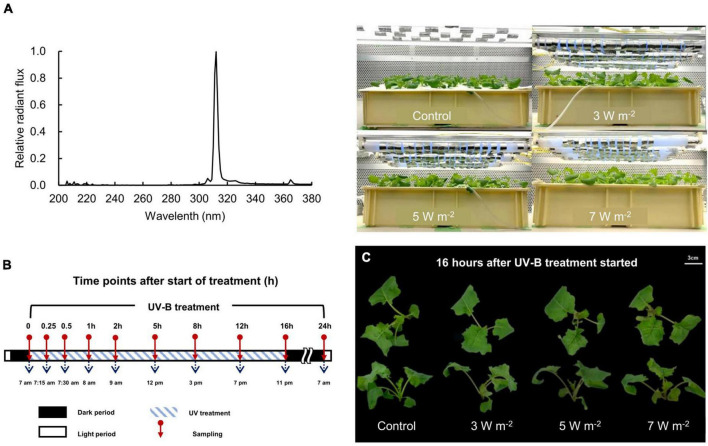
A spectral radiant flux of UV-B lamp (TL20W/01 RS; Philips, Hamburg, Germany) measured with a spectroradiometer (USR-45D; Ushio Inc., Tokyo, Japan) and photographs of canola plants under UV-B lamps **(A)**, UV-B sampling and exposure times **(B)**, and canola plants after 16 h UV-B treatment **(C)**. UV-B irradiation intensity was set to 3, 5, and 7 W m^–2^ by overlapping Al foil on the lamp tube. Sampling was conducted at 0, 0.25, 0.5, 1, 2, 5, 8, 12, 16, and 24 h to investigate time-dependent responses to UV-B irradiation. UV-B irradiation intensities at top of the cultivation panel were set to 3, 5, and 7 W m^–2^. White bar = 3 cm.

The 18-DAS seedlings were subjected to UV-B irradiation at three intensities, including relatively low (intensity 3 W m^–2^; daily dose 259.2 kJ m^–2^), medium (intensity 5 W m^–2^; daily dose 432 kJ m^–2^), and high (intensity 7 W m^–2^; daily dose 604.8 kJ m^–2^) (Figure 1A). The UV-B energy levels used in this study were determined through preliminary experiments. When UV-B levels of 3, 5, and 7 W m^–2^ were applied to 18-DAS canola plants for 3 days, changes in gene expression of *PAL*, *CHS*, *rbcL*, and *rbcS*, and morphological changes such as a decrease in leaf area and increase in specific leaf weight (leaf thickness) were observed in leaves in response to UV-B levels (data not shown). Therefore, the three levels of UV-B used in our study were thought to be effective treatments for inducing both gene expression and bioactive compounds synthesis in canola plants for only 1 day. This study was conducted to confirm the initial response by short-term UV-B irradiation for 1 day. Each UV-B irradiation intensity was regulated by covering the UV-B lamp with Al foil. UV-B irradiation was applied to the canola plants for 24 h. The UV-B irradiation source was a UV-B lamp (TL20W/01 RS; Philips, Hamburg, Germany). Figure 1A shows the spectral radiant flux of the UV-B lamp. The UV-B lamp spectrum was measured with a spectroradiometer (USR-45D; Ushio Inc., Tokyo, Japan). Ten samples were performed during 24 h. The duration of the UV-B treatments and the sampling intervals are shown in Figure 1B. Shoot fresh weights were determined immediately before and after 24 h UV-B treatment.

### Total Phenolic Concentration and Antioxidant Capacity Determination

Canola leaf samples were dried under vacuum in a freezer (FDU-1110; Tokyo Rikakikai Co. Ltd., Tokyo, Japan) at −45°C for 24 h and pulverized in an MM400 ball mill (Retsch GmbH, Haan, Germany) at 20 Hz for 2 min. Dried powder (0.01 g) was extracted with 1 mL of 80% (v/v) acetone. The third leaves of each canola plant that were subjected to different UV-B irradiation intensities were used in the analysis. The UV-B irradiation times were 0, 0.25, 0.5, 1, 2, 5, 8, 12, 16, and 24 h. Total phenolics and antioxidants were extracted from ∼200 mg fresh leaf sample with 80% (v/v) acetone according to the methods of Miller and Rice-Evans (1996) and Ainsworth and Gillespie (2007), respectively. The total phenolic concentration and the antioxidant capacity were determined with a spectrophotometer (V-750; JASCO Corp., Tokyo, Japan) at 765 nm and 730 nm, respectively. Results were expressed as milligrams gallic acid equivalents (GAE) per gram of fresh weight for the total phenolic concentration (GAE mg g^–1^ FW). To determine the antioxidant capacity, acetone extracts were diluted 10-fold, and the results were expressed as millimoles trolox-equivalents per gram fresh weight (TEAC mM g^–1^ FW).

### Flavonoid Concentration Determination

The third leaves of each canola plant were exposed to UV-B irradiation for 0, 0.25, 0.5, 1, 2, 5, 8, 12, 16, or 24 h and were used for this analysis. Approximately, 200 mg of leaf tissue was dried under vacuum in a freezer (FDU-1110; Tokyo Rikakikai Co. Ltd.) at −45°C for 24 h and pulverized in an MM400 ball mill (Retsch GmbH) at 20 Hz for 2 min. About 7 mg dried powder was extracted with 1 ml of 70% (v/v) ethanol and the extracts were sonicated at 30 Hz for 6 min, incubated in the dark at 4°C overnight, and centrifuged at 13,000 × *g* for 2 min at room temperature (20–25°C). The extract (150 μl) was then added to a mixture of 750 μl distilled water plus 45 μl of 5% (w/v) NaNO_2_. The solution was then vortexed and maintained in the dark at room temperature (20–25°C) for 6 min. Then, 90 μl of 10% (w/v) AlCl_3_ was added to the solution, and the mixture was incubated for 5 min. Then, 300 μl of 1 M NaOH and 165 μl of distilled water were added to the solution. The optical density of the reaction mixture was measured in a spectrophotometer (V-750; JASCO Corp.) at 510 nm. The flavonoid concentration was expressed as milligrams catechin equivalents per gram dry weight (mg catechin/g DW).

### Anthocyanin Concentration Determination

Approximately, 400-500 mg fresh second leaf tissue was collected from plants subjected to UV-B irradiation for 0.25, 0.5, 1, 2, 5, 8, 12, 16, or 24 h. The leaf tissue was stored at −80°C until the analysis. The anthocyanins were analyzed according to the method of Mancinelli and Schwartz (1984) with certain modifications. The plant tissues were extracted overnight at 4°C in 400 μl of 1% (v/v) HCl in methanol. Two hundred microliters of distilled water plus 500 μl chloroform were added to the extracts and the mixtures were centrifuged at 13,000 × *g* for 2 min at room temperature (20–25°C). The chloroform layer was separated, 400 μl of the top layer was transferred to a fresh microtube, and 600 μl of 1% (v/v) HCl in methanol was added to it. The anthocyanin concentrations were calculated using the following formula:


(1) A530-0.25×A657

where, the factor 0.25 compensates for the contribution of the chlorophylls to A530. Cyanidin-3-glucoside was a reference standard.

### Hydrogen Peroxide Content Determination

The hydrogen peroxide content of the canola plants was determined according to the method of Velikova et al. (2000). Fresh leaf tissue (0.2 g) was ground twice in the MM400 ball mill (Retsch GmbH) at 30 Hz for 1 min each time. Then 2 mL of 0.1% (w/v) trichloroacetic acid (TCA) was added to the microtube containing the leaf sample. The extract was centrifuged at 12,000 × *g* for 15 min at room temperature (20–25°C). The supernatant (0.5 mL) was added to 0.5 mL of 10 mM potassium phosphate buffer (pH 7.0) plus 1 ml of 1 M KI. The absorbance was measured in a spectrophotometer (V-750; JASCO Corp.) at 390 nm. H_2_O_2_ in the leaf extracts was estimated *via* an equation used to determine standard H_2_O_2_ concentrations. The H_2_O_2_ was expressed as micromoles H_2_O_2_ equivalents per gram fresh weight (μmol H_2_O_2_/g FW).

### Gene Expression Quantification

The third leaves were sampled at 0, 0.25, 0.5, 1, 2, 5, 8, 12, 16, and 24 h UV-B irradiation to investigate time-dependent changes in gene expression. Approximately, 100-150 mg fresh leaf sample was collected and stored at −80°C until the analysis. The RNeasy Plant Mini Kit (Qiagen N.V., Venlo, Netherlands) was used to extract total RNA. The oligonucleotide primers used in the experiments were constructed according to information obtained from the GenBank database (Supplementary Table 1). Complementary DNA (cDNA) was synthesized with a PrimeScript RT Reagent Kit (Perfect Real Time; Takara Bio Inc., Kusatsu, Shiga, Japan) in a GeneAmp PCR System 9700 (Thermo Fisher Scientific, Waltham, MA, United States). The PCR was performed in a Thermal Cycler Dice Real Time System (TP970; Takara Bio Inc.) set to 37°C for 15 min followed by incubation at 85°C for 5 s, termination of the reaction, and cooling at 4°C. TB Green Premix ex Taq (Tli RNaseH Plus; Takara Bio Inc.) was used for the PCR. The PCR conditions for the amplification were 95°C for 5 s (hold), 40 cycles of 95°C for 5 s→60°C for 30 s (two-step PCR), one cycle of 95°C for 15 s→60°C for 30 s→95°C for 15 s (dissociation). The following mRNAs were amplified: phenylalanine ammonia-lyase (*PAL*), cinnamic acid 4-hydroxylase (*C4H*), 4-coumaroyl-CoA ligase (*4CL*), ferulate 5-hydroxylase (*F5H*), chalcone synthase (*CHS*), chalcone isomerase (*CHI*), flavanone 3-hydroxylase (*F3H*), flavonoid 3′-hydroxylase (*F3′H*), flavonol synthase (*FLS*), dihydroflavonol 4-reductase (*DFR*), anthocyanidin synthase (*ANS*), constitutively photomorphogenic (*COP1*), elongated hypocotyl 5 (*HY5*), light-harvesting complex II chlorophyll a/b-binding protein gene (*Lhcb*), ribulose-1,5-bisphosphate carboxylase/oxygenase large subunit (*rbcL*), and ribulose-1,5-bisphosphate carboxylase/oxygenase small subunit (*rbcS*). The mRNA expression levels were normalized against that of the actin (*ACT*) reference gene. Relative gene expression was calculated using the log_2_ treatment:control ratio.

### Microarray Analysis

To explore genome-wide expression changes, samples were selected from plants exposed to 5 W m^–2^ UV-B irradiation for 0.5, 2, 8, 16, and 24 h. Total RNA (1–5 μg) was isolated from each sample with an Agilent Quick Amp Labeling Kit (Agilent Technologies, Palo Alto, CA, United States) and used in the microarray analysis. After fragmentation, the cDNA (1.65 μg) was hybridized using Agilent microarray protocols. The hybridized probes were scanned with an Agilent G4900DA SG12494263. The ratios of normalized fluorescence values were obtained by calculating the log_2_ treatment:control expression ratios.

The differentially expressed genes (DEGs) were used to evaluate differential gene expression of 5 W m^–2^ UV-B irradiation. To analyze reliable data, the noise was excluded (signal evaluation). Flag values of microarray analyzed with Agilent software are as follows: [0] signal was not detected, [1] signal detected but difficult to evaluate, and [2] signal detected. Results of variations in gene expression only involved data with flag values [2]. After data signal evaluation, gene ontology (GO) terms were retrieved and data with confirmed GO function were used for DEGs analysis. Gene differences between those values were calculated based on the Log2 ratio. The result was shown as the number of upregulated (Log2 fold change ≥ 1) and downregulated genes (Log2 fold change ≤ −1).

### Statistical Analysis

The means were subjected to one-way ANOVA in SPSS v. 24 (IBM Corp., Armonk, NY, United States). There were four biological replicates and one plant per replicate. The displayed data are the means and ±SE per treatment. The means were compared using the Tukey–Kramer test. Treatment means were considered significantly different at *p* < 0.05.

## Results

### Growth Characteristics After UV-B Exposure

After 16 h UV-B irradiation exposure, the stems of the canola plants turned red. Stem redness intensified with UV-B irradiation intensity (Figure 1C). At higher UV-B irradiation intensity levels, the fresh weight of the canola plant decreased after 24 h. However, none of the UV-B treatments (3, 5, or 7 W m^–2^) notably affected canola growth relative to the control after 24 h (Supplementary Figure 1).

### Total Phenolic, Flavonoid, and Anthocyanin Concentrations, and Antioxidant Capacity in Response to UV-B

**FIGURE 2:**
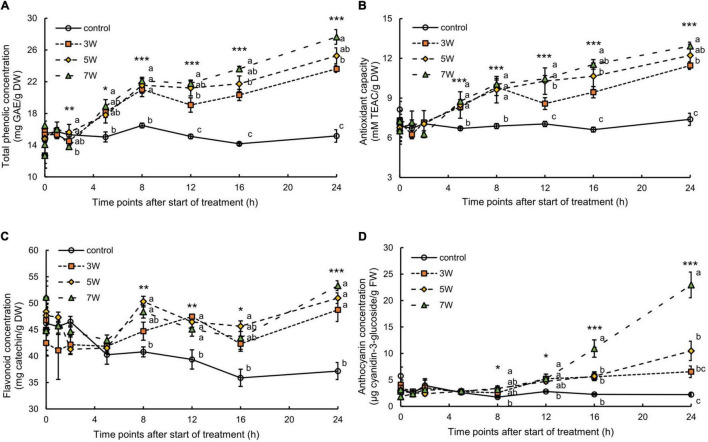
Time courses of total phenolic concentration **(A)**, antioxidant capacity **(B)**, flavonoid concentration **(C)**, and anthocyanin concentration **(D)** in canola subjected to 3, 5, and 7 W m^–2^ UV-B irradiation intensity. Third (total phenolic and antioxidant capacity and flavonoid) and second (anthocyanin) leaves from the bottom of each canola plant were subjected to various UV-B irradiation intensities. Vertical bars indicate SE (*n* = 4). Different letters (a, b, and c) indicate a significant difference using Tukey–Kramer test (*p < 0.05, **p < 0.01, and ***p < 0.001, respectively).

The total phenolic concentration and antioxidant capacity of the canola leaves varied with UV-B exposure time and irradiation level (Figure 2). The total phenolic concentration increased 2 h after the onset of UV-B irradiation and continuously increased over time (Figure 2A). The plants subjected to 3, 5, and 7 W m^–2^ UV-B irradiation for 16 h showed a 1.43-, 1.53-, and 1.66-fold increase, respectively, in total phenolic concentrations compared with the control plants. The total phenolic concentration increased with UV-B irradiation intensity, showing a 1.82-fold increase in the plants subjected to 7 W m^–2^ UV-B irradiation for 24 h compared with the control. After 24 h, the plants exposed to 7 W m^–2^ UV-B irradiation presented a 1.75-fold increase in antioxidant capacity compared with the control plants (Figure 2B).

The total flavonoid concentration in the canola leaves varied with UV-B irradiation intensity and exposure time (Figure 2C). The total flavonoid concentration increased 8 h after the onset of UV-B irradiation and continuously increased. They reached their maxima after 24 h UV-B irradiation but did not significantly differ among UV-B irradiation intensity levels.

The plants subjected to 3, 5, and 7 W m^–2^ UV-B irradiation showed 2.59-, 2.63-, and 5.15-fold increase, respectively, in anthocyanin concentrations after 16 h of the treatment compared with the control plants (Figure 2D). The plants exposed to 7 W m^–2^ UV-B irradiation for 24 h showed an 11.21-fold increase in anthocyanin concentrations compared with the control plants. The stems and leaves of the canola plants turned red after 16 h UV-B exposure.

### Hydrogen Peroxide Production in Response to UV-B

**FIGURE 3:**
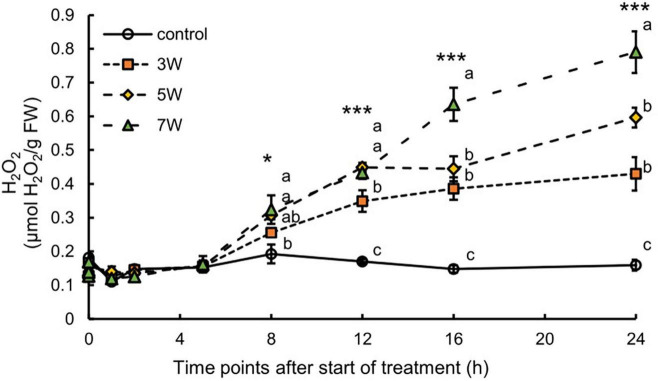
Time courses of H_2_O_2_ content in canola subjected to 3, 5, and 7 W m^–2^ UV-B irradiation intensity. Second leaves from the bottom of each canola plant were subjected to various UV-B irradiation intensities. Vertical bars indicate SE (*n* = 4). Different letters (a, b, and c) indicate a significant difference using Tukey–Kramer test (*p < 0.05, **p < 0.01, and ***p < 0.001, respectively).

In this work, the association between H_2_O_2_ content and plant UV-B exposure was like that for anthocyanin (Figure 3). The H_2_O_2_ content significantly increased 8 h after the onset of UV-B irradiation and continuously increased. The plants subjected to 7 W m^–2^ UV-B irradiation for 24 h showed maximum H_2_O_2_ content, representing a 4.97-fold increase compared with the control plants.

### Expression of Genes Related to UVR8-Dependent UV-B Signaling Pathway

**FIGURE 4:**
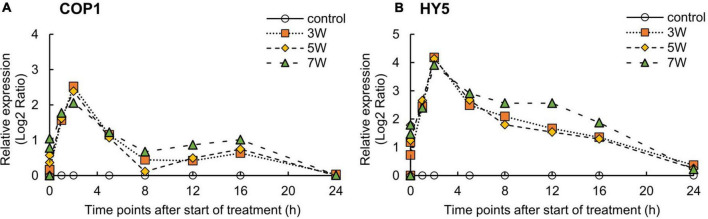
Time courses of *COP1* **(A)** and *HY5* **(B)** mRNA expression in canola plants. Vertical bars indicate SE (*n* = 4). Third leaves from the bottom of each canola plant were subjected to various UV-B irradiation intensities. UV-B irradiation intensities at the top of the cultivation panel were set to 3, 5, and 7 W m^– 2^. Line graphs indicate log_2_ fold changes (treatment:control) in gene expression levels.

The expression levels of *COP1* and *HY5* were significantly induced by UV-B irradiation (Figure 4). The levels of *COP1* and *HY5* were upregulated after the onset of UV-B irradiation and reached maxima at 2 h; thereafter, *COP1* and *HY5* levels were substantially downregulated.

### Expression of UV-B-Responsive Genes Related to Phenylpropanoid and Flavonoid Biosynthesis

To identify DEGs from microarray data, genes were determined to be differentially expressed in the UV-B samples compared with the control samples (Supplementary Figure 2). GO annotation was used to functionally analyze the canola plant. Approximately, 2,500 gene transcripts were assessed after the signal evaluation was applied during 24 h of UV-B exposure (Supplementary Figure 2). Differential gene expression analysis showed a total of 291, 398, 197, 192, and 305 at 0.5, 2, 8, 16, and 24 h of UV-B exposure, respectively. Among them, 188 and 181 upregulated genes showed significantly highest fold changes at 0.5 and 2 h of UV irradiation. The number of downregulated genes was 217, showing the highest fold change at 2 h of UV irradiation.

Table 1 shows the results of the microarray analyses of the variations in the expression levels of the genes related to phenylpropanoid and flavonoid biosynthesis, respectively. The relative gene expression patterns identified using RT-PCR corroborated the variations in gene expression revealed by microarray analysis. *PAL* was continuously expressed between 2 h and 16 h UV-B irradiation. The expression levels of *C3′H* and *CCoAOMT* were significantly upregulated after 2 h UV-B exposure. *F5H* expression was upregulated after 8 h UV-B treatment. The downstream *SGT* expression was upregulated throughout the UV-B irradiation period. The expression levels of all flavonoid genes, except *FLS* and *ANS*, continuously varied between 2 and 16 h of UV-B treatment (Table 1).

**TABLE 1:**
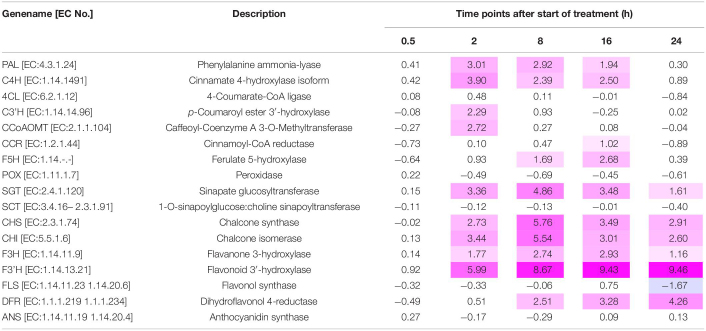
Gene expression variation related to phenylpropanoid and flavonoid biosynthesis in canola subjected to UV-B irradiation.

*Values indicate log_2_ ratios (n = 1) obtained by calculating treatment:control gene expression ratios. UV-B irradiation intensity at the top of the cultivation panel was set to 5 W m^–2^.*

*Positive values that increased by ≥ 1.0 are in pink. To analyze reliable data, the noise was excluded (signal evaluation). Flag values of microarray analyzed with Agilent software are as follows: [0] signal not detected, [1] signal detected difficult to evaluate, and [2] signal detected. Results of variations in gene expression only involved data with flag values [2].*

**FIGURE 5:**
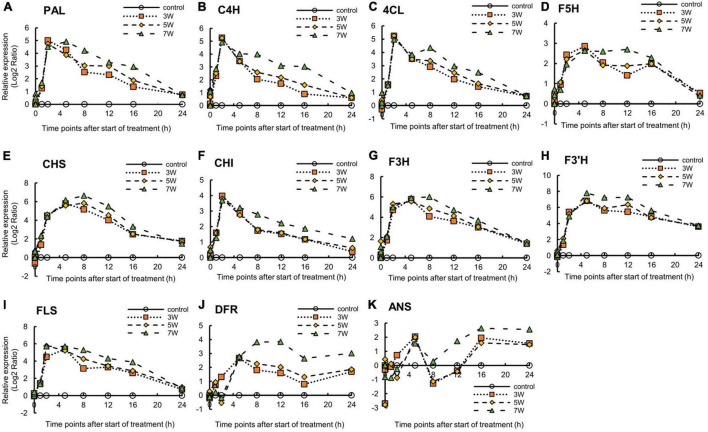
Time courses of *PAL* **(A)**, *C4H* **(B)**, *4CL* **(C)**, *F5H* **(D)**, *CHS* **(E)**, *CHI* **(F)**, *F3H* **(G)**, *F3’H* **(H)**, *FLS* **(I)**, *DFR* **(J)**, and *ANS* **(K)** mRNA expression in canola (*n* = 4). Third leaves from the bottom of each canola plant were subjected to various UV-B irradiation intensities. UV-B irradiation intensities at top of the cultivation panel were set to 3, 5, and 7 W m^–2^. Line graphs indicate log_2_ fold changes (treatment:control) in gene expression levels.

The expression levels of the genes encoding secondary metabolites and the shikimate pathway were analyzed (Supplementary Figure 3). Relative gene expression varied with UV-B irradiation intensity and exposure time. The *PAL* and *C4H* expression levels reached maxima after 2 h UV-B treatment (Figure 5). *PAL* and *C4H* encode the first and second key enzymes in the phenylpropanoid pathway. In the plants subjected to 7 W m^–2^ UV-B irradiation, *PAL* and *C4H* expression levels were downregulated at a slower rate than in the plants subjected to the other UV-B irradiation intensity levels. The trend in *4CL* expression in response to UV-B irradiation resembled those for *PAL*, *C4H*, and *F5H* expression, which peaked after 5 h UV-B treatment and increased once again after 16 h UV-B exposure. The plants exposed to 7 W m^–2^ UV-B irradiation maintained high *F5H* expression levels between 5 and 12 h UV-B exposure. *CHS* expression reached maxima at 5 h (3 W m^–2^) and 8 h (5 and 7 W m^–2^) UV-B treatment and decreased thereafter. *CHI* expression reached a peak after 2 h at all the UV-B irradiation intensity levels and decreased thereafter. *F3H* expression reached a peak at 5 h after UV-B treatment and decreased thereafter. *F3’H* expression reached a maximum after 5 h UV-B irradiation and remained at high levels between 5 and 12 h UV-B treatment. *FLS* expression reached peaks at 2 h (5 and 7 W m^–2^) and 5 h (3 W m^–2^) UV-B exposure and decreased thereafter. *DFR* expression reached a maximum at 5 h UV-B exposure and decreased to a minimum after 16 h. In the plants subjected to 5 and 7 W m^–2^ UV-B irradiation, *DFR* expression reached maxima after 5 h UV-B treatment and decreased thereafter. *ANS* expression showed a trend similar to that of *DFR* only until 5 h UV-B treatment. At all UV-B irradiation intensities, *ANS* expression was dramatically downregulated after 8 h and was upregulated thereafter.

### Expression of Genes Related to Photosynthesis in Response to UV-B Radiation

**FIGURE 6:**
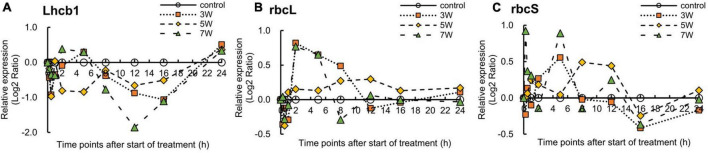
Time courses of **(A)** *Lhcb1*, **(B)** *rbcL*, and **(C)** *rbcS* mRNA expression in canola (*n* = 4). Third leaves from the bottom of each canola plant were subjected to various UV-B irradiation intensities. UV-B irradiation intensities at top of the cultivation panel were set to 3, 5, and 7 W m^–2^. Line graphs indicate log_2_ fold changes (treatment:control) in gene expression levels.

Figure 6 shows the log_2_ ratios of the expression levels of the photosynthesis-related genes, namely *Lhcb1*, *rbcL*, and *rbcS*, within 24 h of UV-B irradiation. Relative *Lhcb1* expression was reduced under all UV-B treatments. However, the timing of the decrease in *Lhcb1* expression varied with UV-B irradiation intensity. Nevertheless, all UV-B irradiation intensities lowered gene photosynthesis-related gene expression after 8 h UV-B treatment. Then, *Lhcb1* expression was upregulated after 16 h UV-B exposure.

The *rbcL* expression levels varied with UV-B irradiation intensity. For 3 and 7 W m^–2^ UV-B irradiation treatments, *rbcL* expression was rapidly upregulated after 2 h, downregulated after 8 h, and showed expression levels similar to those of the control thereafter.

The *rbcS* expression levels also varied with UV-B irradiation intensity. For 3 and 7 W m^–2^ treatments, *rbcS* expression was rapidly upregulated after 4 h and downregulated after 8 h. Under the 5 W m^–2^ UV-B treatment, however, *rbcS* expression was immediately upregulated at 8 h and downregulated thereafter. Nevertheless, the variations in the expression levels did not significantly differ among photosynthesis-related genes (data not shown).

## Discussion

### Effect of UV-B Irradiation on Growth

The growth of the canola plants was not significantly affected by short-term (24 h) UV-B treatment (Supplementary Figure 1). Ravindran et al. (2010) reported that UV-B exposure for 2 days (20 W m^–2^, 2 h per day; daily dose 144 kJ m^–2^) did not affect the growth parameters (shoot length, fresh weight, dry weight, or leaf area) of *Indigofera tinctoria*, whereas 4-day UV-B irradiation reduced growth. When tobacco was subjected to various UV-B irradiation doses (0, 37, 740, 1,480, and 2,960 J m^–2^) for 4 days, cell proliferation significantly declined. Cell death was also significantly induced after 4 days of UV-B irradiation at all intensities (Takahashi et al., 2015). These results indicate that exposure to 3, 5, and 7 W m^–2^ UV-B irradiation for 24 h did not adversely affect canola plant growth. These treatments were not intense enough to cause permanent oxidative damage to the plants. However, long-term UV-B treatment may adversely affect plant growth, and the responses to it may vary with plant species, developmental stage, and leaf thickness (Piluzza and Bullitta, 2011; Wang et al., 2018).

### Effects of UV-B Irradiation on Bioactive Compounds and Hydrogen Peroxide

High-intensity UV light can damage DNA, proteins, and the photosynthetic apparatus, including the chloroplasts. These injuries can adversely affect plant growth and development (Diffey, 1991). However, low-intensity UV irradiation may induce various protective mechanisms in plants, including the accumulation of low-molecular-weight compounds, such as antioxidants that suppress oxidative damage and maintain redox homeostasis (Bharti and Khurana, 1997). UV-B irradiation can induce the biosynthesis of phenylpropanoids and flavonoids that absorb UV (Jenkins, 2009; Robson et al., 2015). In the leaf epidermis, phenolics reduce oxidative damage and protect the photosynthetic apparatus by inhibiting the penetration of UV-B irradiation into the inner photosynthetic layers (Agati and Tattini, 2010).

Here, short-term UV-B irradiation at 3, 5, and 7 W m^–2^ activated secondary metabolite biosynthesis pathways and promoted antioxidant accumulation in canola plants (Figures 2, 3). The concentrations of secondary metabolites, such as total phenolics, antioxidants, total flavonoids, anthocyanins, and H_2_O_2_, significantly increased in response to all UV-B exposure levels (Figures 2, 3). These results suggested that the stems and leaves of canola plants turned red because the concentration of anthocyanins was significantly increased by UV-B irradiation; thus, anthocyanin expression may be associated with higher UV-B irradiation levels (Figure 2D). Furthermore, the results of total phenolic and antioxidant capacity showed almost similar trends, which agreed with results of previous studies that showed a positive linear relation between total phenolic content and antioxidant capacity (ABTS and DPPH) (Javanmardi et al., 2003; Pennycooke et al., 2005; Piluzza and Bullitta, 2011).

Based on the homeostasis between ROS and antioxidants, ROS is continuously generated in different cellular compartments as by-products of various metabolic pathways, such as plant respiration and photosynthesis, even under normal conditions (Berni et al., 2019). A constant adjustment of ROS concentration is achieved by non-enzymatic secondary metabolites such as CAT, APX, GPX, and GR; therefore, plants have a normal metabolism (Kapoor et al., 2015). However, when plants were subjected to a stressful environment (biotic and abiotic), an imbalance between ROS and antioxidants occurs, resulting in a rapid increase in ROS concentration and inducing irreversible oxidative processes such as cell death by “oxidative burst” (Sharma et al., 2012). However, the appropriate concentration of ROS acts as a signaling molecule participating in important developmental and physiological processes and responds to highly generated ROS by generating secondary metabolites such as polyphenol, terpene, and vitamins (Sharma et al., 2012; Xia et al., 2015). In particular, H_2_O_2_ plays a central role in signaling pathways because of its relatively long lifetime and can freely diffuse across membranes through aquaporins (Bienert et al., 2007; Møller et al., 2007). Moreover, at the appropriate levels, ROS may act as signaling molecules in various intracellular processes and induce antioxidant biosynthesis.

In this study, the concentration of H_2_O_2_ showed a tendency to increase as energy increased, but all UV treatments did not significantly affect plant growth and morphological changes. Therefore, the amount of ROS generated was not high enough to inhibit canola growth and was within the appropriate range. The observed increases in the bioactive compound content at 3, 5, and 7 W m^–2^ UV-B irradiation indicated that these radiation levels were within a suitable range to stimulate antioxidant biosynthesis in canola. Previous studies reported that the UV levels they used increased the bioactive compound content in plants (Lois, 1994). *Arabidopsis* plants were exposed to various levels of UV-B irradiation intensity (0.10, 0.15, 0.24, 0.37, and 0.8 W m^–2^) for 30 h and flavonoid accumulation was the highest at 0.15 W m^–2^ UV-B irradiation (Lois, 1994). However, exposure to high UV levels may actually decrease the bioactive compound content by impairing cellular function. Similar results were obtained for sweet basil leaves exposed to various levels of UV-B irradiation intensity (0, 2.3, 3.6, and 4.8 W m^–2^) (Ghasemzadeh et al., 2016). The concentrations of total phenolics, total flavonoids, and individual flavonoids, and phenolic acids were the highest at 3.6 W m^–2^ UV-B irradiation but significantly declined at 4.8 W m^–2^ UV-B irradiation. In *Medicago sativa*, antioxidant and flavonoid compounds significantly increased in response to lower UV-B irradiation levels (17.35 μW cm^–2^ d^–1^), whereas higher UV-B irradiation levels caused severe damage and adversely affected growth and development (Gao et al., 2019). The foregoing results suggest that, below certain UV exposure thresholds, the content of bioactive compounds, such as flavonoids, may be proportional to the UV dose. In this study, there is a possibility that 7 W m^–2^ UV-B irradiation was the optimal UV-B exposure level, whereas 10 W m^–2^ reduced the bioactive compound content in canola.

### Effects of UV-B Irradiation on Expression of the Secondary Metabolites Biosynthesis-Related Genes

The expression of the genes encoding antistress and antioxidant compounds is regulated by the UVR8 pathway (Hideg et al., 2013). The cytoplasmic UVR8 photoreceptor is an active dimer and it becomes a monomer during UV-B absorption. UV-B-induced UVR8 photoreceptor monomers directly reacted with E3 ubiquitin ligase “constitutively photomorphogenic 1” (COP1) and promoted “elongated hypocotyl 5” (HY5) transcription in *Arabidopsis* nuclei (Brown et al., 2005; Favory et al., 2009; Wu et al., 2012). In *Arabidopsis*, the accumulation of HY5 transcripts promoted flavonoid biosynthesis by activating chalcone synthase (CHS) and prevented UV-B absorption by epidermal tissue (Bharti and Khurana, 1997; Kliebenstein et al., 2002; Brown and Jenkins, 2008). Photomorphogenic responses, such as an increase in leaf thickness, axillary branching, and the induction of UV-absorbing compounds, are mediated by the activation of UV-B photoreceptor UV “resistance locus 8” (UVR8) in response to low-to-moderate UV-B irradiation levels (0.1-21 μmol m^–2^ s^–1^ and 1.77-1.07 W m^–2^) (Hectors et al., 2007; Brown and Jenkins, 2008; Rizzini et al., 2011). *COP1* and *HY5* transcription were induced in *Arabidopsis* by low UV-B flux rates (0.1 μmol m^–2^ s^–1^) (Brown and Jenkins, 2008). In this study, the expression levels of *COP1* and *HY5* were upregulated immediately after UV-B exposure and attained the highest expression levels after 2 h. UV-B-absorbing compounds were also affected by UVR8 pathway activation and accumulated in the canola plants (Figure 2). However, high UV-B irradiation levels may induce ROS biosynthesis, which damages DNA, proteins, membranes, and so on, and impedes plant growth and development (Brown and Jenkins, 2008; Hideg et al., 2013). Here, the UV-exposed plants were not harmed, and their bioactive compound content had significantly increased. These results suggest that the canola plants were subjected to only low-to-moderate levels of UV-B irradiation.

The genes *PAL, C4H, 4CL, F5H, CHS, CHI, F3H, F3’H, FLS, DFR*, and *ANS* are key genes in the phenylpropanoid and flavonoid biosynthesis pathways. *PAL* leads to the main bifurcation in phenylpropanoid metabolism and is an upstream gene. *F3’H, DFR*, and *ANS* are downstream genes in flavonoid biosynthesis. The times to peak expression differed among genes; genes with downregulated expression required a long time to reach maximum expression (Figure 5); however, with variable gene expression (Table 1), the trends in upregulation were like those encoding phenylpropanoids (*PAL*, *C4H*, and *F5H*) and flavonoids (*CHS*, *CHI*, *F3H*, *F3’H*, and *DFR*) according to qRT-PCR (Figure 5). When *Chrysanthemum morifolium* was exposed to UV-B irradiation for different durations, *HY5* expression was rapidly upregulated after 1 h, whereas the expression levels of *CHS, CHI, F3H*, and *FLS* increased after 6 h (Yang et al., 2018). Similar trends were reported in a previous study analyzing *CHS, CHI, F3H, DFR*, and *ANS* expression in radish sprouts (Su et al., 2016). *CHS, CHI*, and *F3H* are located upstream in the flavonoid biosynthesis pathway, and their expression levels were upregulated after 12 h UV-B exposure. However, the expression levels of downstream *DFR* and *ANS* were upregulated after 24 h UV-B irradiation. Moreover, the expression levels of phenylpropanoid and flavonoid-related genes were consistently upregulated at 7 W m^–2^ UV-B irradiation. At 7 W m^–2^ UV-B irradiation, gene expression levels decreased at a slower rate than at 3 and 5 W m^–2^ UV-B irradiation. Therefore, 7 W m^–2^ UV-B irradiation stimulated gene expression more effectively than 3 or 5 W m^–2^, regardless of the duration of UV-B exposure.

Microarray analysis was used for the whole-genome exploration of gene expression profiles in canola plants. Quantitative RT-PCR results supported the microarray data. In the microarray analysis, many DEGs, down- or upregulated, were observed during 24 h of UV-B irradiation (Supplementary Figure 2). These genes identified in this study have been implicated in secondary metabolite syntheses (Table 1). The DEGs were significantly upregulated at the 0.5 and 2 h of UV-B exposure relative to the other time point. The significantly upregulated DEGs with unknown genes were from the 0.5 and 2 h of UV-B irradiation. These results suggested that DEGs were involved in the complex molecular mechanisms necessary for resistance to UV irradiation in canola plants. In addition, even in the quantitative RT-PCR results (Figures 4, 5), genes of the UVR8 pathway and the upper group of the secondary metabolite rapidly increased after 2 h of UV exposure. The DEGs results indicated that numerous genes in UV-B treated plants have undergone molecular biological changes on 0.5 and 2 h of UV-B irradiation. Even at 0.5 h, the number of upregulated genes was large. It is possible that unanalyzed (or unconfirmed) genes were expressed and ultimately led to upregulation.

The time points at which the expression levels of genes were upregulated varied with plant species. UV-B irradiation induced genes related to phenylpropanoid and flavonoid biosynthesis, which increased polyphenol antioxidant content. The results of the gene expression analyses were consistent with those for the characteristics of the bioactive compounds (Figure 2). The observed increases in the bioactive compound content in plants subjected to 3, 5, and 7 W m^–2^ UV-B irradiation indicated that all UV-B exposure levels were within a range suitable to stimulate antioxidant phenolic compound biosynthesis in canola. All UV-B treatments increased the production of these bioactive compounds by upregulating the expression levels of several key genes in the biosynthetic pathways for these compounds. However, there were significant time lags between the observed increases in the levels of these compounds and upregulation of the expression levels of genes encoding them. The expression levels of *PAL, C4H*, and *4CL* were dramatically increased after 2 h UV-B irradiation. The relative expression of *F5H* reached a peak after 5 h UV-B irradiation (Figure 5). The total phenolic concentration coincided with the peaks in *F5H* expression and significantly increased, starting at 5 h UV-B irradiation (Figure 2A). The flavonoid biosynthesis genes (*CHS, F3H, F3’H*, and *FLS*) were induced in response to HY5 transcript accumulation following UV-B irradiation, and their peak expression levels occurred after 5-8 h UV-B irradiation. The expression levels of *ANS* and *DFR* reached their maxima at 12-16 h UV-B treatment. According to a previous report, some genes involved in the biosynthesis of the phenylpropanoid pathway are not expressed continuously when exposed to UV-B irradiation, but genes are expressed rapidly for a short period and then return to the basal level (Höll et al., 2019; Meyer et al., 2021). Our results also showed a tendency to decrease to the basal level after each gene expression peaked.

Total flavonoid concentration and relative anthocyanin content increased at 5-8 h and 12-16 h UV-B irradiation, respectively (Figure 2). Regardless of the time points at which the expression levels of the gene reached their maxima, the bioactive compound content continued to rise. These reactions may have been defense responses in anticipation of future exposure to the same abiotic stress. If UV irradiation is interrupted, the plant may use its “memory capacity” to continue increasing the bioactive compound content (Bruce et al., 2007). Time differences between gene expression and bioactive compound biosynthesis were reported for wheat leaves under drought stress (Ma et al., 2014). Their *CHS, CHI, F3H, FLS, DFR*, and *ANS* expression levels reached their maxima at 12-16 h after UV. Timely application of short-term, moderate-intensity UV-B irradiation to leafy vegetables 1-2 days preharvest might enhance antioxidant phytochemical production without yield or quality loss in horticultural crops raised in plant factories and vertical farms under artificial light. Thus, bioactive compound biosynthesis might have continued to increase in canola even after the 24-h UV-B treatment.

### Effects of UV-B Irradiation on Expression of the Photosynthetic Metabolites Biosynthesis-Related Genes

The photosynthesis-related genes *Lhcb1, rbcL*, and *rbcS* were differentially expressed throughout the entire UV-B irradiation period (Figure 6). The *Lhcb1, rbcL*, and *rbcS* expression levels in pea plants were reduced by UV-B treatment (Strid et al., 1994). *Lhcb1* encodes the chlorophyll a/b-binding protein of the photosystem (PS) - light-harvesting antenna complex and maintains the photosynthetic apparatus (Andersson et al., 2003). *Lhcb1* also modulates stomatal movement and promotes plant stress tolerance (Xu et al., 2012). In this study, *Lhcb1* expression was rapidly downregulated under all UV-B treatments after 8 h and upregulated after 24 h UV-B treatment (Figure 6A). These results suggest that short-term UV-B exposure had a negative effect on the photosynthetic machinery. Kalbina and Strid (2006) reported that *Lhcb1* mRNA expression was reduced in *Arabidopsis* by short-term UV-B irradiation. O_2_^⋅–^ and H_2_O_2_ are secondary messengers involved in the downregulation of the expression of photosynthetic genes, such as *Lhcb* (Kalbina and Strid, 2006). Hence, the generated ROS might have downregulated *Lhcb1* expression in this study.

The CO_2_ fixation is achieved through photosynthesis, and the enzyme Rubisco is involved in this complex process. It was reported that this highly sensitive mechanism may be inhibited by UV-B irradiation (Fedina et al., 2010). The rbcS (Rubisco small subunit) and rbcL (Rubisco large subunit) proteins are susceptible to degradation. However, the expression levels of *rbcS* and *rbcL* are upregulated to compensate for the damage caused to their protein products by oxidative stress (Xiong et al., 2010). Here, *rbcS* expression was upregulated after 2 h and downregulated after 8 h UV-B treatment (Figure 6C); however, *rbcL* expression was upregulated after 4 h and downregulated after 16 h UV-B treatment. The times of peak expression differed slightly between *rbcS* and *rbcL.* Nevertheless, the rbcS and rbcL protein levels were drastically downregulated by UV-B treatment. It is therefore possible that rbcS and rbcL mRNA levels were rapidly upregulated to regenerate the lost photosynthetic proteins (Xiong et al., 2010). Enhanced gene transcription might compensate for ROS-mediated protein damage/loss and help maintain the photosynthetic machinery subjected to UV-B irradiation. Increased *rbcS* and *rbcL* transcription have also been reported to cause drought and salinity stress, which promote oxidative stress (Lilley et al., 1996; Fu et al., 2007). Thus, *rbcS* and *rbcL* expression levels were rapidly upregulated to compensate for the decreases in photosynthetic protein content caused by UV-B exposure. Nevertheless, prolonged UV-B exposure might delay or even suppress upregulation of *rbcS* and *rbcL* expression.

In canola, short-term UV-B irradiation downregulated the expression of genes implicated in photosynthesis but did not adversely affect plant growth (Supplementary Figure 1). However, if UV-B irradiation continues for > 2 days, plant growth would significantly decrease.

## Conclusion

This study demonstrated that the genes regulating secondary metabolite biosynthesis in canola were affected by UV-B irradiation intensity and duration. UV-B irradiation for ≤ 24 h was merely a mild stressor for canola and did not damage its photosynthetic machinery or hinder its growth. To minimize UV-B-induced damage, phenylpropanoid and flavonoid biosynthesis were rapidly activated, and antioxidant phytochemicals accumulated. The expression levels of the genes governing targeted secondary metabolic pathways significantly differed with UV-B exposure duration. There were also temporal differences between gene expression and bioactive compound accumulation. The concentrations of all antioxidants increased in response to the peak expression levels of the genes regulating the phenylpropanoid and flavonoid pathways. The plants subjected to 7 W m^–2^ UV-B irradiation showed 1. 82-, 1. 75-, and 11.21-fold increase in total phenolic, flavonoid, and anthocyanin concentrations, respectively, compared with the unexposed controls because of the inherent “memory mechanism” of the plant. To the best of our knowledge, this work is the first to demonstrate a temporal difference between the accumulation of antioxidants and the induction of the genes encoding them in UV-B-treated canola plants. Based on the discoveries in this work, timely application of short-term, moderate-intensity UV-B irradiation to canola and other leafy vegetables at 1-2 days preharvest might enhance the biosynthesis of health-promoting antioxidants without compromising crop yield or quality.

As biosynthesis and accumulation of bioactive compounds are regulated by complex temporal and spatial patterns, more in-depth studies are needed on temporal differences between gene expression and target bioactive compounds’ accumulation. Short-term UV irradiation could be a widely used technique for controlling the activation timing of target compounds in plant factories and vertical farms.

## Data Availability Statement

The datasets presented in this study can be found in online repositories. The names of the repository/repositories and accession number(s) can be found at: https://www.ebi.ac.uk/arrayexpress/E-MTAB-11034.

## Author Contributions

J-HL and SS: performance of experiments, sample collection, and analyses of chemical data. J-HL: writing – original draft preparation. EG: writing – review and editing, conceptualization, experimental design, supervision, and funding acquisition. All authors read and agreed to the final version of the manuscript.

## Conflict of Interest

The authors declare that the research was conducted in the absence of any commercial or financial relationships that could be construed as a potential conflict of interest.

## Publisher’s Note

All claims expressed in this article are solely those of the authors and do not necessarily represent those of their affiliated organizations, or those of the publisher, the editors and the reviewers. Any product that may be evaluated in this article, or claim that may be made by its manufacturer, is not guaranteed or endorsed by the publisher.
