# Supplementary material for: Time-Course of Changes in Photosynthesis and Secondary Metabolites in Canola (Brassica napus) Under Different UV-B Irradiation Levels in a Plant Factory With Artificial Light
Source: Front Plant Sci. 2021 Dec 22;12:786555. doi: 10.3389/fpls.2021.786555 (PMC8730333; doi:10.3389/fpls.2021.786555)
Supplement: Supplementary file 1 [file Table_1.docx]

**Supplementary Table S1. Primers and characteristics of genes used in RT-PCR**.

| Gene symbol | Gene name |  | Primer sequence (5’-3’) | Product length (bp) |
| --- | --- | --- | --- | --- |
| *ACT* | Actin | Forward  Reverse | AGTACTCTTCCAGCCGTCGC  GCGCCGTGATCTCTTTGCTC | 182 |
| *COP1* | Constitutively photomorphogenic | Forward  Reverse | ACAACCACCGCCACGAAAAC  GGCTGCAACAGGGACAATCG | 198 |
| *HY5* | Elongated hypocotyl 5 | Forward  Reverse | GGAGGACTCCGGCTGAGAAA  TAGAACCACCGCTTCCCCTC | 243 |
| *PAL* | Phenylalanine ammonia-lyase | Forward  Reverse | TCTACACGTACGCGGACGAC  TAGGTAGCACCGCCTTGAGC | 171 |
| *C4H* | Cinnamic acid 4-hydroxylase | Forward  Reverse | CTGGCGCAAGATGAGGAGGA  TGGTCGCGGAGTCAGGATTC | 134 |
| *4CL* | 4-Coumarate-CoA ligase | Forward  Reverse | TGTTCCGCCGCTTGTGATTG  ACACTGGTCCTGCCTCTGTC | 185 |
| *CHS* | Chalcone synthase | Forward  Reverse | CTGCGGCCCAGACCATCTTA  ATCTTCTCCGCCTTGAGCCC | 250 |
| *CHI* | Chalcone isomerase | Forward  Reverse | TTGCTCTCTCCCCTAACGGC  CCCAGGAGACACACCCTTCT | 146 |
| *F3H* | Flavanone 3-hydroxylase | Forward  Reverse | TCGCTCGAGACTTCTTCGCC  AGCCAAACCCATCAGCCTCT | 242 |
| *FLS* | Flavonol synthase | Forward  Reverse | CGTCGACCTAAGCAACCCCA  GGAGTCTTCTGGCTTCGCGA | 193 |
| *F3’H* | Flavonoid 3'-Hydroxylase | Forward  Reverse | GCGGTTCCTTTGGTTGTGCA  TCCACCGTGCAAGACCTAGC | 112 |
| *DFR* | Dihydroflavonol 4-reductase | Forward  Reverse | AGCAGCTTGGGATTACGCGA  CCCTTGGCAGCAGCTTGTTC | 231 |
| *ANS* | Anthocyanidin synthase | Forward  Reverse | TCAGACCGCTGTAACCACCG  GGTCCCCGAGCTGTTGAAGT | 209 |
| *F5H* | Ferulate 5-hydroxylase | Forward  Reverse | CGTCAGCGATAGAGTGGGCA  TCCACACGTCGGTCAAGTCC | 106 |
| *Lhcb1* | Light-harvesting complex Ⅱ chlorophyll a/b-binding protein gene | Forward  Reverse | CCATCTGGCAGCCCATGGTA CAAGCATGGCCCATCTGCAG | 193 |
| *rbcL* | Ribulose-1,5-bisphosphate carboxylase/oxygenase large subunit | Forward  Reverse | ACTGCGGGTACATGCGAAGA  TGCATTGCACGGTGGATGTG | 167 |
| *rbcS* | Ribulose-1,5-bisphosphate carboxylase/oxygenase small subunit | Forward  Reverse | CATTCCCAGTCACCCGCAAG GTGGCCACACCTACGAAACG | 249 |

Symbols, names, designed primer sequences, and product lengths (base pairs) indicated for selected genes searched by Blast among *Brassica napus* sequences in GenBank database.
