# Supplementary material for: Time-Course of Changes in Photosynthesis and Secondary Metabolites in Canola (Brassica napus) Under Different UV-B Irradiation Levels in a Plant Factory With Artificial Light
Source: Front Plant Sci. 2021 Dec 22;12:786555. doi: 10.3389/fpls.2021.786555 (PMC8730333; doi:10.3389/fpls.2021.786555)
Supplement: Supplementary file 2 [file Data_Sheet_1.docx]

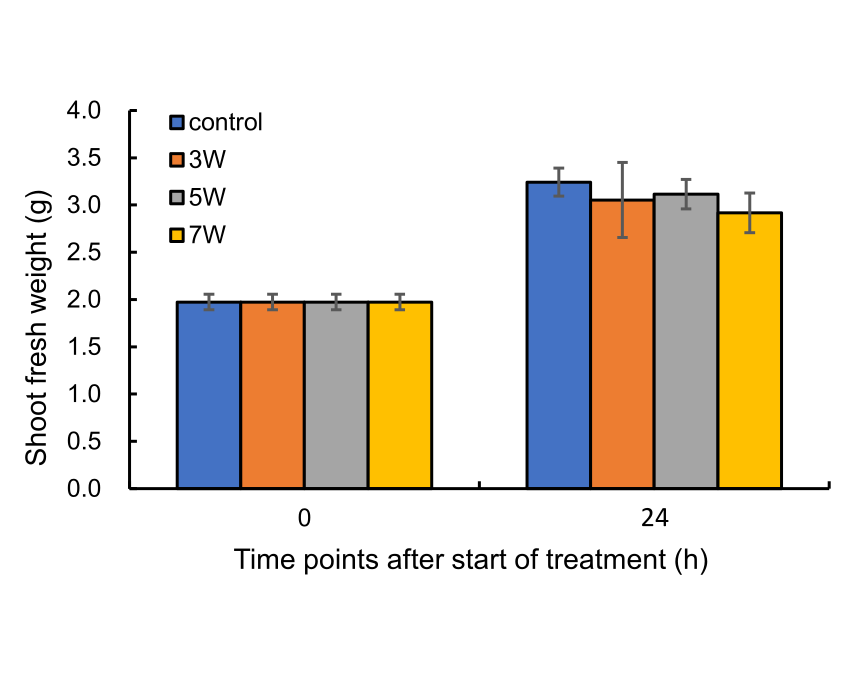


**Supplementary Figure S1.** **Shoot fresh weight before and 24 h after UV-B treatment onset**. UV-B irradiation intensities at top of cultivation panel were set to 3, 5, and 7 W m^-2^.
