# Supplementary material for: Time-Course of Changes in Photosynthesis and Secondary Metabolites in Canola (Brassica napus) Under Different UV-B Irradiation Levels in a Plant Factory With Artificial Light
Source: Front Plant Sci. 2021 Dec 22;12:786555. doi: 10.3389/fpls.2021.786555 (PMC8730333; doi:10.3389/fpls.2021.786555)
Supplement: Supplementary file 3 [file Data_Sheet_2.docx]

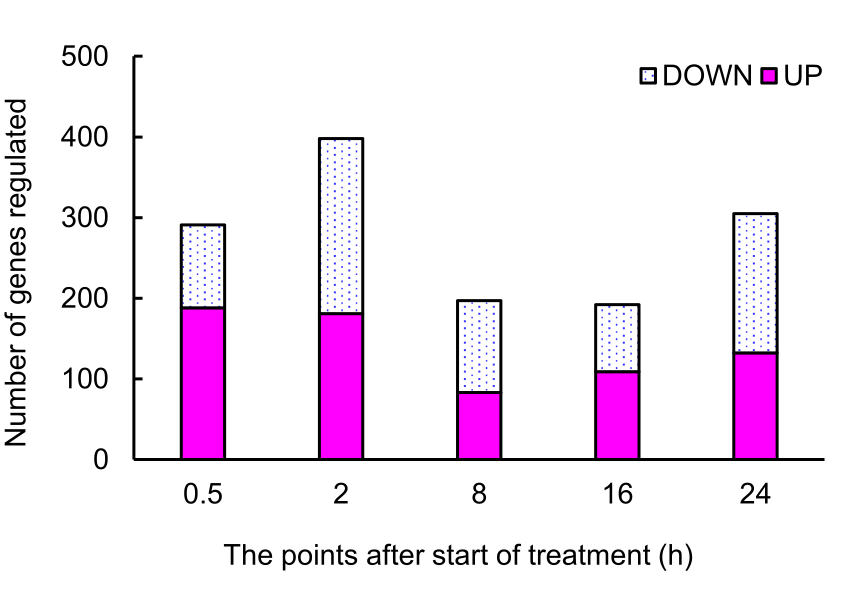


**Supplementary Figure S2.** **Total numbers of differentially upregulated and downregulated genes in canola irradiated with UV-B.** The UV-B irradiation intensity at the top of the cultivation panel was set to 5 W m^−2^. The numbers of upregulated (Log2 fold change ≥ 1) and downregulated-genes (Log2 fold change ≤ -1) are presented.
